# What Were the Motives?

**Published:** 1900-12

**Authors:** 


### WHAT WERE THE MOTIVES?

Upon another page will be found an article under the heading, "What does it mean," by Dr. Arrington, of Goldsboro, N. C., in which he takes as a text a quotation from the *Items of Interest* in regard to the motives actuating the men who closed the career of the American Dental Association and on its ruins formed the present National Dental Association.

The quotation in question says, "The idea of having subdivisions of the Association, to be known as Eastern, Western, and Southern branches, it is well known now was placed in the constitution as a means of inducing the Southern men to agree to union."

Space will not permit a full review of the history of that work, but as the writer was an active and interested participant in it, he can assure both Dr. Arrington and the veracious chronicler in the *Items of Interest* that the motive for the change from the American to a National Association was simply that it had been deemed unwise to continue two, so-called, national organizations, one for the North and the other for the South, and that this opinion was not confined to any one section of the country. The North made a great sacrifice towards harmony in giving up absolutely the American. The South still retained the Southern as a branch. The bonds that bound the members to either association could not be lightly severed. These, however, were not weighed seriously in the balance, and the National Dental Association was organized.

The writer had something to do with the original draft of the constitution, and while not satisfied with it as a whole, he made no captious opposition; neither was the final adoption of that instrument in accordance with his ideas, but at no time could the motives of his colleagues be impugned. The whole effort, from first to last, *was for the good of the dental profession of the United States,* and no one had anything to gain by seeking an opposite course.

The question of the formation of Eastern, Western, and Southern branches of the National body was a more difficult problem.

The writer can answer only for the Eastern. The committee having that in charge met and considered the subject. It could not see its way clear at that time to form an organization as contemplated by the constitution of the National. It was, therefore, postponed to a more satisfactory period. It was very evident that the formation then of a branch would have been exceedingly ill-advised. The National Association was itself on trial, and there were grave doubts as to its permanency. Happily these fears have been dispelled by three successful meetings. It is possible that ultimately the organization of the contemplated branches may be perfected, but it must be apparent that there are now altogether too many dental associations. With the increase in local, State, and interstate associations, there is a growing tendency to professional weakness. Dentistry is too narrow to admit of such wide extension, and, it must be evident, proper support would not be forthcoming. The opinion of the writer is that we should strengthen those associations now in existence before any attempt is made to organize others.

If the writer in the *Items of Interest* erred through ignorance, he may be pardoned, but if with malicious intent to stir up ill feeling, it cannot be too severely condemned. In any event, it has not a particle of truth to recommend it or to make it worthy the space given.

